# Eye Movements in Darkness Modulate Self-Motion Perception

**DOI:** 10.1523/ENEURO.0211-16.2016

**Published:** 2017-01-25

**Authors:** Ivar Adrianus H. Clemens, Luc P. J. Selen, Antonella Pomante, Paul R. MacNeilage, W. Pieter Medendorp

**Affiliations:** 1 Donders Institute for Brain, Cognition, and Behaviour, Radboud University Nijmegen, 6525 HR, Nijmegen, The Netherlands; 2 German Center for Vertigo and Balance Disorders, Ludwig Maximillians University Hospital of Munich, D-81377 Munich, Germany

**Keywords:** integration, oculomotor, perception, self-motion, vestibular

## Abstract

During self-motion, humans typically move the eyes to maintain fixation on the stationary environment around them. These eye movements could in principle be used to estimate self-motion, but their impact on perception is unknown. We had participants judge self-motion during different eye-movement conditions in the absence of full-field optic flow. In a two-alternative forced choice task, participants indicated whether the second of two successive passive lateral whole-body translations was longer or shorter than the first. This task was used in two experiments. In the first (*n* = 8), eye movements were constrained differently in the two translation intervals by presenting either a world-fixed or body-fixed fixation point or no fixation point at all (allowing free gaze). Results show that perceived translations were shorter with a body-fixed than a world-fixed fixation point. A linear model indicated that eye-movement signals received a weight of ∼25% for the self-motion percept. This model was independently validated in the trials without a fixation point (free gaze). In the second experiment (*n* = 10), gaze was free during both translation intervals. Results show that the translation with the larger eye-movement excursion was judged more often to be larger than chance, based on an oculomotor choice probability analysis. We conclude that eye-movement signals influence self-motion perception, even in the absence of visual stimulation.

## Significance Statement

Multiple sensory signals have been identified to contribute to our estimate of self-motion. We show that eye-movement signals, even in complete darkness, influence self-motion perception.

## Introduction

An accurate estimate of self-motion is important to guide interactions with the environment. During passively induced motion, both vestibular and optic flow signals provide information about self-motion (Gibson et al., 1955; Benson et al., 1986; Israël and Berthoz, 1989; Harris et al., 2000; Angelaki and Hess, 2005; Chen et al., 2010; Carriot et al., 2013). These signals drive perception, but they also drive compensatory eye movements that work to maintain fixation on world-stationary objects. The associated oculomotor signals could also contribute to self-motion estimates because they are correlated with head displacement. While many studies have shown that the brain uses eye-movement signals to extract the optic flow component related to self-motion (Warren and Hannon, 1988; Royden et al., 1992; Freeman and Banks, 1998; Lappe et al., 1999), a direct influence of eye movements on self-motion perception has not been tested.

When gaze is world stable during whole-body translation, eye displacement correlates with translation size and is modulated by fixation depth (Schwarz et al., 1989; Paige et al., 1998; McHenry and Angelaki, 2000; Medendorp et al., 2002). In contrast, when fixation is body fixed the eyes remain stationary in their orbits (Paige et al., 1998; Ramat et al., 2005), making them no longer informative about self-motion. Nevertheless, the brain may assume that eye movements are always informative about self-motion, as during the linear vestibulo-ocular reflex (LVOR), leading it to equate the absence of eye movements to a cue indicating the absence of self-motion. The brain can integrate the eye-movement cue with vestibular and other sensory cues to derive a weighted estimate of self-motion. If so, self-motion with body-fixed gaze should be underestimated compared with self-motion with a world-fixed gaze, despite identical vestibular cues. In addition, if oculomotor signals are always integrated with vestibular signals to estimate self-motion, the effects of eye movements should be observable even during movement in complete darkness. In particular, unconstrained eye movements induced by the LVOR, which will have magnitudes between body-fixed and world-fixed fixation, should parametrically relate to the perceived self-motion.

To test whether eye movements are used in self-motion perception, we used a two-alternative forced choice (2-AFC) paradigm in which participants were presented with two consecutive lateral translations. They had to indicate whether the second translation was longer or shorter than the first. Eye movements during each interval were constrained using either a world-fixed or body-fixed fixation point or were not constrained at all (i.e., free). We show that identical translations were perceived to be shorter when gaze was body fixed compared with world fixed. Furthermore, using a basic linear weighting model, we predicted perceived displacement during the free-gaze condition based on the weighted integration of vestibular signals and the unconstrained eye-movement magnitude. In an additional experiment, we show that natural variations in eye-movement magnitude, without a fixation constraint, correlate with the perceived translation magnitude. We conclude that the brain includes oculomotor signals in computations contributing to self-motion perception, even in the absence of optic flow or other visual stimulation.

## Materials and Methods

### Participants

Eighteen naive participants (9 female), between 19 and 36 years of age, provided written informed consent to participate in the experiments. Eight participants performed the first experiment, and 10 performed the second experiment. All participants were free of any known vestibular or neurological disorder and had normal or corrected-to-normal visual acuity. Participants never received any feedback about their performance.

### Experimental setup

A motorized linear sled (Clemens et al., 2012) was used to laterally translate participants following a minimum jerk profile (Flash and Hogan, 1985) of fixed duration (1 s) and amplitudes ranging from 1 to 27 cm. Participants were seated on the sled such that the interaural axis aligned with the motion axis. They were restrained using a 5 point seat belt and a chin rest. The head was held in place using a sled-fixed mold that resembled headphones and pressed on the head surrounding the pinna. Auditory cues were suppressed using white noise presented through in-ear headphones. Experiments were conducted in complete darkness except for visual fixation points, which were projected by a laser pointer on a black bar 50 cm in front of the participant at eye level. Laser pointers used to project body-fixed targets were attached to the sled. Those used to project world-fixed targets were mounted on the wall behind the sled.

Eye movements were recorded at 500 Hz using either an EyeLink II (first experiment) or an Eyelink 1000 system (second experiment; both systems are from SR Research). Cameras were mounted to the sled and therefore remained stable with respect to the head during the entire experiment. Because the head and body positions were fixed during the experiment, the orientation of the eyes within the head, as measured by the trackers, was equivalent to the orientation of the eyes in space. The eye-tracking systems were calibrated before each session using 11 evenly spaced calibration points ranging from −22° to 22°. We used linear regression to link camera coordinates to gaze angles.

### Experiments

We conducted two separate experiments. In the first, we determined the point of subjective equality (PSE) when comparing translations under world-fixed and body-fixed fixation and without stable fixation. In the second experiment, we removed all fixation constraints and investigated the influence of acceleration-induced eye movements on translation perception.

### Experiment 1: perceived self-motion across different fixation conditions

We used a 2-AFC task to measure perceived linear self-motion across the following three different fixation types: world-fixed, body-fixed, and unconstrained (free) fixation. We refer to these as world, body, and free, respectively. A trial contained two sequential translation intervals of equal duration (1 s) and in the same direction (either leftward or rightward). Different fixation types were presented in the two translation intervals. Participants were instructed to judge whether the translation during the second interval was longer or shorter compared with the first interval. They were additionally instructed to always look at the fixation point when it was visible; no instructions were given for when the fixation point was switched off (i.e., during free fixation).

**Figure 1.**
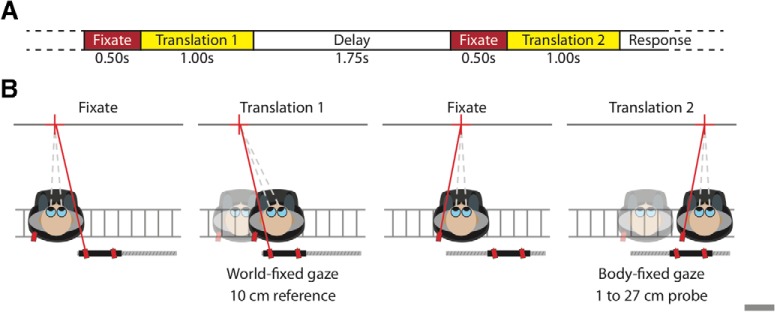
***A***, Time course of key events within a single trial. In each of the two intervals, a 0.50 s fixation period (red) precedes the lateral translation (yellow). A 1.75-s-long delay period (shown in white) separates the two intervals. After the second translation, the participant responded whether this second translation was longer or shorter than the first. ***B***, Top view of the setup illustrating key events during a rightward body-world trial where the world-fixed reference interval was presented first. The condition tested is marked using an asterisk in Table 1. The sled-fixed as well as the world-fixed lasers (red) used to present the fixation targets on a black bar (dark gray bar) that runs in parallel with the sled track (light gray bars). First panel, Participant fixates the world-fixed target (red cross) at the start of the first interval. Second panel, Translation with world-fixed fixation target. Third panel, Body-fixed fixation at the start of the second fixation interval. Fourth panel, Translation with body-fixed fixation in second interval.

The time evolution of a single trial is shown in Figure 1. Each trial started with the onset of a central fixation point (i.e., aligned between the eyes) for 0.5 s. Subsequently, the first translation interval commenced. Depending on the fixation type, the fixation point remained visible (world and body) or was extinguished (free) during the translation interval. The trial shown in Figure 1 depicts the 10 cm reference translation with world fixation. After this first interval, a delay followed in which the participant was kept in complete darkness for 1.75 s. Then, the central fixation point reappeared, followed 0.5 s later by the second interval, in which the probe translation was presented. The set of possible probe translations ranged from 1 to 27 cm in equidistant steps of ∼0.4 mm. The fixation type in the probe interval was always different than that in the associated reference interval (the trial in Fig. 1 illustrates body fixation). After the second interval, the participant had to indicate whether he or she perceived the second translation as longer or shorter than the first using a one-dimensional joystick. Moving the joystick away from the body indicated that the second movement was longer, while moving it toward the body indicated that the second movement was shorter.

Thus, a trial consists of two translations with different fixation types. In the three main conditions, we compare the body versus world, world versus free, and body versus free-fixation types. For each main condition, we varied the fixation type that served as the reference stimulus and the order in which reference and probe were presented, which gives a total of four variations per main condition (Table 1). In addition, we varied translation direction (either leftward or rightward on consecutive trials). The amplitude of the probe translation was adaptively chosen using the Psi method. This method picks the amplitude for the next trial that maximizes the expected decrease in entropy based on participants’ responses to earlier trials (Kontsevich and Tyler, 1999). This was done separately for all 24 trial types (3 main conditions × 2 reference stimuli × 2 reference/probe orders × 2 translation directions; Table 1). A total of 25 trials was collected per trial type, yielding a total of 200 trials for each of the three main conditions.

**Table 1:** List of the three main comparisons that we tested in experiment 1

Comparison	Reference	First translation	Direction
Body vs world	Body	Reference	Right
			Left
		Probe	Right
			Left
	World	Reference	Right*
			Left
		Probe	Right
			Left
Body vs free	Body	Reference	Right
			Left
		Probe	Right
			Left
	Free	Reference	Right
			Left
		Probe	Right
			Left
World vs free	World	Reference	Right
			Left
		Probe	Right
			Left
	Free	Reference	Right
			Left
		Probe	Right
		Left

The (10 cm) reference movement was presented in either the first or second movement interval. We also manipulated movement direction (leftward or rightward), yielding a total of 24 trial types.

*Condition shown in Figure 1*B*.

Trials were presented in three 1 h sessions. To prevent dark adaptation, we turned on the lights for 5 s after every block of six trials, and for at least 30 s every four blocks. We made sure that each of the 24 unique trial types was presented once every four blocks. After each block, the adaptive procedure determined which translation amplitudes to test in the following block. To increase the number of data points available to the adaptive psychometric procedure at the beginning of the experiment, we collapsed across translation direction and reference order for the first 10 trials of every condition. After those collapsed trials, the procedure ran separately for each of the 24 distinct trial types.

### Experiment 2: perceived self-motion under free eye movement

We used a 2-AFC task to measure perceived linear self-motion under free eye movement in both intervals. A trial contained two sequential translation intervals of equal duration (1 s) and in the same direction (either leftward or rightward). Participants were instructed to judge whether the translation during the second interval was longer or shorter compared with the first interval.

Each trial started with the onset of a fixation point displayed either at a visual azimuth angle of −10°, 0°, or 10° from the mid-sagittal plane. The fixation point remained on for 0.75 s and disappeared as soon as the sled movement started. After the first movement interval, a delay followed in which the subject was kept in complete darkness for 0.25 s. Then the second fixation point, with the same eccentricity as the first, appeared for 0.75 s. Once the fixation point extinguished, the second movement interval started. After this second interval, the subject indicated whether the second translation was perceived for a longer or shorter time compared with the first interval. The subjects did not receive any instruction on gaze requirements once the fixation point turned off.

A trial consisted of a 10 cm reference translation preceded or followed by a probe translation. The set of possible probe translations ranged from 7 to 13 cm in equidistant steps of 1.5 cm, resulting in five different reference/probe comparisons. On 50% of the trials, the two translations had equal amplitudes of 10 cm, whereas the other four reference/probe comparisons were equally distributed in the remaining 50% of the total trials. Subjects conducted a total of 480 trials in a single session of ∼1 h.

### Data analyses

#### Experiment 1

To analyze the results of the first experiment, we quantified the perceived probe translation for each condition and reference/probe sequence (Table 1) by calculating the probability of the probe translation judged longer compared with the 10 cm reference translation as a function of actual probe translation, given by *x*. We used a maximum-likelihood fit of a cumulative Gaussian function to summarize the psychometric data, as follows:

(1) $$P\left(x\right)=\lambda +\left(1-2\lambda \right)\frac{1}{\sigma \sqrt{2\pi }}\overset{x}{\underset{-\infty }{\int }}e^{-{\left(\left|x\right|–PSE\right)}^{{/}_{2\sigma^{2}}2}},dy$$

in which |x| represents the size of the absolute probe displacement. The mean of the Gaussian represents the PSE. The slope of the curve reflects the precision (1/σ) of reference probe discrimination performance. Parameter *λ*, representing the lapse rate, accounts for stimulus-independent errors caused by subject lapses or mistakes and was restricted to small values (*λ* < 0.06). Fits were performed using the Psignifit toolbox (Wichmann and Hill, 2001a,b).

For each trial type (Table 1), we also quantified the eye-movement magnitude. We first discarded trials containing blinks as well as trials in which the final eye position exceeded two SDs from the average of the condition. Based on these criteria, 6.1%, 3.6%, and 1.6% of all trials, respectively, were rejected based on errors in body, world, and free fixation. In addition, we rejected 1.2% of all trials because participants blinked within the movement interval.

For the remaining trials, we computed the average ratio between the measured eye excursion, $\varphi_{i}$, and the angle that would be needed were the trial testing the world-fixed condition. The latter is computed by taking the arc-tangent of the actual translation distance, $m_{i}$, divided by the fixation depth, $d_{i}$, which for small values of φ can be approximated by g=φm/d. We computed this ratio, g, for every fixation type and interval (Table 1). Ideally, for body-fixed trials g=0, and for world-fixed trials g=1. Using this ratio, we are able to compute the expected eye excursion, ${\hat{\varphi }}_{m}=gd/m$, for any given translation distance, even those we did not explicitly measure.

Using a simple cue integration model, we investigated whether intersubject and intercondition differences in the observed PSEs in conditions containing a translation under free fixation depend on actual eye-movement behavior. We modeled perceived distance, *p*, as a weighted linear combination of a vestibular and an oculomotor estimate of translation (Eq. 2). We assumed that the vestibular estimate is equal to the actual translation, *m*, and that the oculomotor estimate is equal to expected eye movement, given the actual, ${\hat{\varphi }}_{m_{i}}$. As the weights represent the relative contributions of the oculomotor and vestibular systems, together they sum to 1 in Equation 2. Thus, the weighting parameter, *α*, regulates the eye-movement contribution and 1−α regulates the vestibular contribution, as follows:

(2) $$p=\alpha {\hat{\varphi }}_{m}d+\left(1-\alpha \right)m=\alpha gm+\left(1-\alpha \right)m$$

By definition, the probe displacement is perceived as equal in length to the 10 cm reference displacement at the PSE. By substituting both sides by the right-hand side of Equation 3 and using subscripts for reference (*r*) and probe intervals (*p*), we obtain the following:

(3) $$\alpha g_{r}m_{r}+(1-\alpha )m_{r}=\alpha g_{p}m_{p}+(1-\alpha )m_{p}+\varepsilon$$

In the experiment, the reference displacement, $m_{r}$, was always 10 cm, and the probe displacement, $m_{p}$, was equal to the measured PSE for the presented combination of fixation types (i.e, PSE in Eq. 1). This model (i.e., Eq. 3) was then fit to data from the body and world conditions using linear regression, finding weight α that minimizes the sum of squared errors $\left(\sum^{\text{​}}\varepsilon^{2}\right)$, as follows:

(4) $$m_{r}-m_{p}=\alpha (g_{f_{p}}m_{p}-g_{f_{r}}m_{r}+m_{r}-m_{p})+\varepsilon$$

By only using data from conditions where a visual fixation point was present (i.e., body vs world) to fit the model, we examined whether the same weight α also explains the PSEs found in the conditions where fixation was free. To this end, we solved Equation 3 for $m_{p}$ and computed PSE estimates, $P\hat{S}E$, for the body versus free and world versus free conditions (Eq. 5), as follows.

(5) $$m_{p}=P\hat{S}E=\frac{\alpha g_{r}+\left(1-\alpha \right)}{\alpha g_{p}+\left(1-\alpha \right)}m_{r}$$

#### Experiment 2

To analyze the results of experiment 2, we quantified the relationship between the eye displacement and subjects’ perceptual responses by computing choice probabilities using receiver operating characteristic (ROC) analysis (Britten et al. 1996; Uka and DeAngelis 2004). We discarded trials in which >50% of eyelink samples were missing during any of the two movement intervals due to blinks. Also, trials in which the eye positions during the last 25 samples of the fixation were >2 SDs apart prior to the first and second translation interval were discarded. Based on these criteria, 12% of all trials were rejected because the subject blinked, and 5% of all trials were rejected because participants did not follow the fixation constraints. We further assumed that the slow-phase eye movements are compensatory for the motion and that that quick phases and saccades are corrective, catch-up eye movements. Following standard approaches (Wyatt, 1998), the latter were detected on a trial-by-trial basis by first finding all of the peaks in the velocity signal (i.e., all direction changes of the velocity signal). Samples around these peaks were removed (−7 to +7 ms) from the velocity trace. From the remaining samples, the slow-phase eye velocity trace was reconstructed by temporal integration. This trace was integrated once more to compute the eye-movement magnitude.

Next, we computed the difference between the eye-movement magnitudes of the two consecutive translations (Δeye; see Fig. 6*A*). For each reference/probe comparison, the distribution of Δeye across trials was normalized using *z*-scores, and these *z*-scores were pooled across all reference/probe comparisons (see Fig. 6*B*). Subsequently, we computed the choice probability (CP) based on the *z*-scored Δeye. First, we separated the trials into two distributions based on the perceptual response (“second longer” vs “second shorter”), and subsequently we constructed an ROC curve from these distributions and derived the choice probability value as the area under the ROC curve.

## Results

The first experiment investigates the influence of fixation type and associated eye movements on the perception of self-motion. Participants were presented with two subsequent lateral translations (Fig. 1), and they had to judge whether the second was longer or shorter than the first. During each interval, participants fixated a body-fixed or world-fixed target (body and world fixation) or were moved in absence of a fixation point (free fixation).

**Figure 2.**
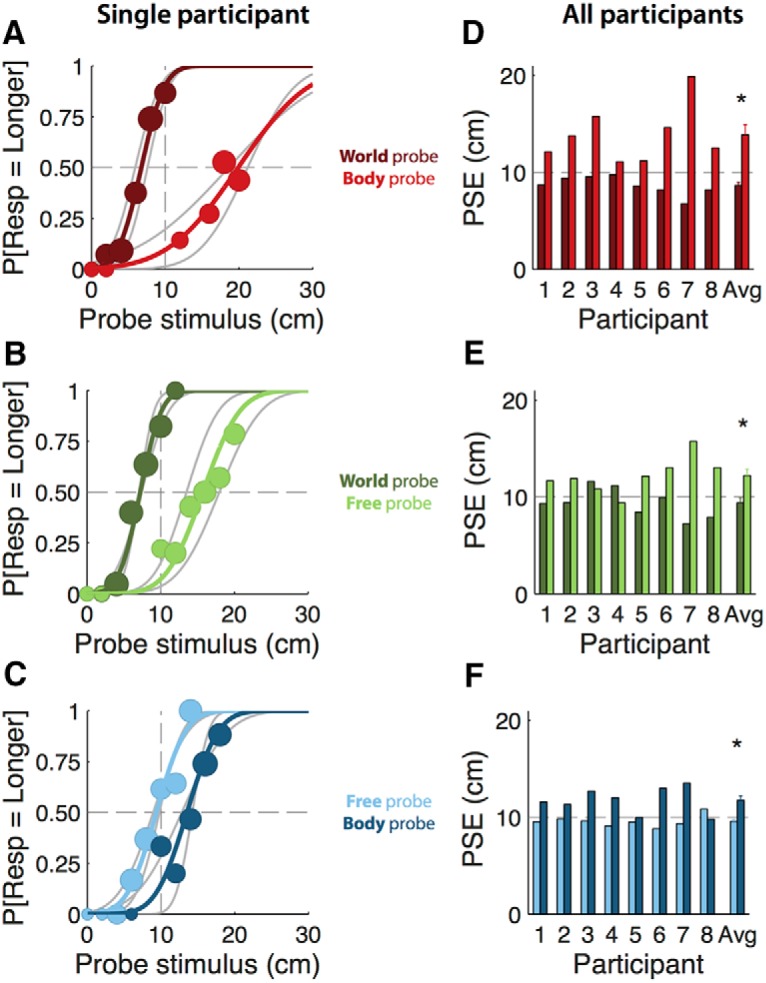
***A–C***, Psychometric curves (colored lines) and associated binned data (circles) for participant number 7 (top row). Circle size represents the number of trials within each 2 cm bin. Binning was only performed in order to visualize this participant’s responses and was not used otherwise. Gray lines show psychometric curves before collapsing across reference order. Dashed gray lines represent the 10 cm reference movement. ***A***, Body–world comparison; body reference, dark red; world reference, light red. ***B***, World–free comparison; world reference, light green; free reference, dark green. ***C***, Body–free comparison; body reference, dark blue; free reference, light blue. ***D–F***, PSEs for all participants and the average ±SE (bottom row). Dashed gray lines represent the 10cm reference movement. Colors are as in ***A–C***. ***D***, Body–world comparison. ***E***, World–free comparison. ***F***, Body–free comparison. Because a *t* test revealed a main effect of reference order (*t*_(47)_ = −5.2, *p* < 0.01), we used the mean PSE across reference order (e.g., colored lines) instead of the PSE without collapsing across reference order (e.g., gray lines); these values were not significantly different.

The performance of one participant is illustrated in the left column of Figure 2. Each row shows one main condition: body versus world fixation (top/red), world versus free fixation (middle/green), and body versus free fixation (bottom/blue). The lighter and darker colors in each panel indicate which fixation type was the reference movement (Fig. 2, legend). The shift of the psychometric functions relative to the 10 cm reference (i.e., the PSE) quantifies the influence of fixation type. For example, the rightward shift of the light red curve in Figure 2*A* means that for a body fixation a longer translation (∼19 cm) was required for that translation to be perceived, equivalent to a 10 cm reference translation with world fixation. On the other hand, the leftward shift of the dark red curve means that a shorter translation with world fixation (∼7 cm) was required for that translation to be perceived equivalent to the 10 cm reference translation with body fixation. Together, these oppositely directed shifts demonstrate that translations with world fixation were perceived longer than equivalent translations with body fixation, regardless of which translation was the reference. Similarly, the shifts in Figure 2*B* show that world-fixation translations were also perceived to be longer than free-fixation movements, and Figure 2*C* shows that free-fixation translations were perceived to be longer than body fixation translations. Note that Figure 2 also shows an effect on slope, which will be further discussed in the section “Precision depends on PSE.”

Similar results were obtained for all subjects, as shown by the individual PSEs for all participants (Fig. 2, right column). Statistical significance of the fixation-induced effects for each main condition (Fig. 2*D*, world vs body, *E*, world vs free, *F*, free vs body) was evaluated by comparing PSEs between the two reference conditions using a paired *t* test. These PSEs were significantly different in all cases (world vs body, *t*_(7)_ = −4.09, *p* < 0.05; world vs free, *t*_(7)_ = −2.48, *p* < 0.05; free vs body, *t*_(7)_ = −3.38, *p* < 0.05). As for the example subject, these results indicate that translations made with body fixation are perceived for a shorter period than with world fixation. This could mean that self-motion perception is modulated by eye movements, even in the absence of full-field optic flow, or that self-motion perception is modulated by the presence of a small visual fixation point. The latter explanation is refuted by the free-fixation translations, which account for possible confounds of the small fixation point and were perceived longer than body-fixation intervals and shorter than free-fixation translation intervals. This result would be expected if the eye-movement gain is <1 but >0 during the free-fixation intervals.

**Figure 3.**
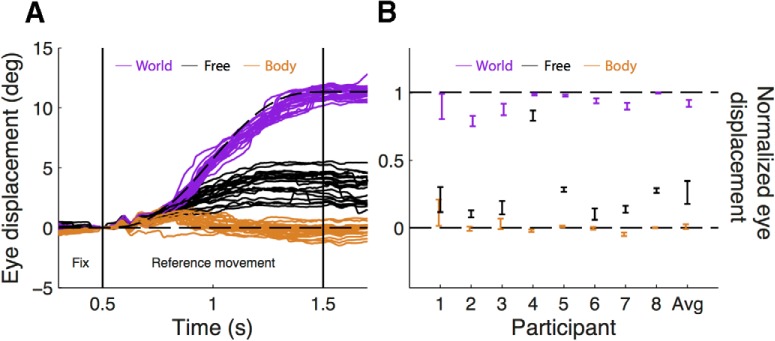
***A***, Actual (solid lines) eye-movement traces of one participant during world fixation (purple), body fixation (brown), and free fixation (black). For the body and world fixation, the ideal traces are indicated by the dashed lines. All traces shown are for 10 cm reference movements. ***B***, Normalized eye position for each participant (±95% confidence interval) at the end of the translation interval (error bars) for world fixation (purple), body fixation (brown), and free fixation (blue). In addition, the average ±SE across all participants is shown. Zero indicates that the eyes remained stationary relative to the body, and 1 indicates that eye position was perfectly world fixed.

In order to relate psychophysical performance to eye-movement behavior, we recorded and analyzed eye movements during both intervals of every trial for all subjects. Exemplar eye traces for the 10 cm reference translation for the three fixation types are depicted in Figure 3*A*. Fixation behavior was quite accurate for both body fixations, where no eye movements were expected, and world fixation, where eye-movement excursions of ∼11º were expected, occasionally supported by corrective saccades. Under free-fixation, eye-movement magnitude was intermediate between body and world fixation and magnitudes were more variable. A similar pattern was observed in all participants, as illustrated by the normalized eye-movement data (see Materials and Methods; Fig. 3*B*).

**Figure 4.**
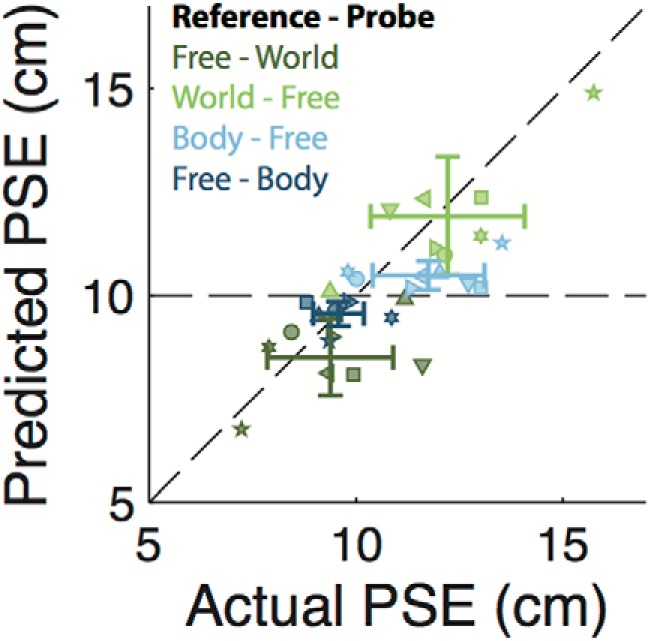
Eye movement-based prediction for the PSE plotted against the actual PSE. A data point (symbol) is shown for each participant (symbol shape) and condition (symbol color) pair, following the same color scheme as in Figure 2. The identity line, corresponding to a perfect prediction, is shown in black.

**Figure 5.**
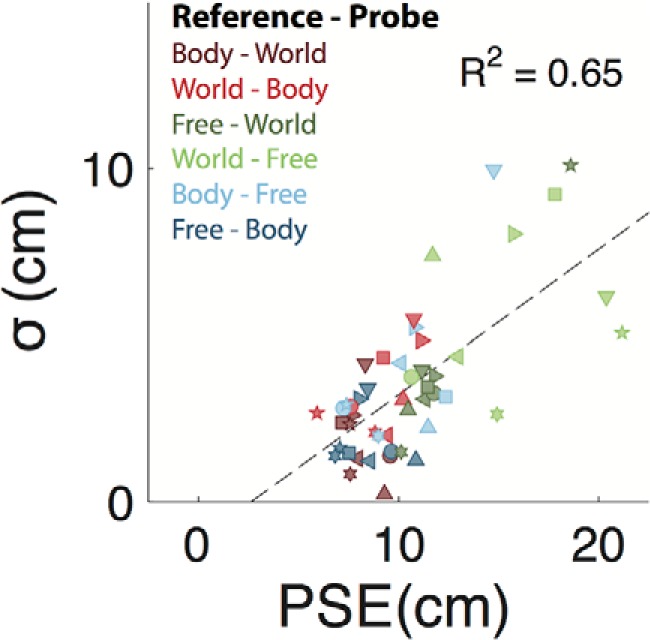
Relationship between PSE and response uncertainty (σ). A data point is shown for every participant (symbol) and condition (color) pair. Same color scheme as in Figure 2. The dashed black line is the linear regression trend line (*R*^2^ = 0.65).

To quantify the role of eye movements in self-motion perception, we tested a linear model in which perceived translation is a weighted average of a vestibular estimate (equal to the actual translation) and an oculomotor estimate (equal to the normalized eye-movement times the actual translation; Eq. 2). This model contains a single free parameter (α), which corresponds to the relative weight given to the oculomotor estimate. We fitted this model to the body versus world conditions and obtained the value of the oculomotor weight for every subject (Tables 2, 3). The average (±SD) oculomotor weight is 0.25 ± 0.12, indicating that the relative contribution of the eye-movement signal to the self-motion estimate is ∼25%. Note that participant 4, whose oculomotor weight is furthest from this mean (*α* = 0.06), also shows a radically different eye-movement gain during the free fixation (Fig. 4*B*). We then used these oculomotor weights along with the normalized eye movement values to predict the PSEs in the remaining four conditions according to Equation 5. The predicted PSEs are plotted against the actually observed PSEs in Figure 5. The positive correlation (*ρ* = 0.78, *p* < 0.01) between observed and predicted PSEs suggests that eye movements are indeed used in self-motion perception, even in the absence of a fixation point (i.e., during free fixation). Furthermore, the fact that data points generally cluster near the unity line shows that our simple model does reasonably well in predicting perceptual performance across subjects and conditions based on oculomotor weight and normalized eye-movement magnitude only. This holds true even for subject 7 whose oculomotor weight (Table 2) was approximately double the average, yet whose data points remain close to the unity line.

**Table 2:** Estimated eye movement contribution (α) to the perception of self-motion (see Eq. 5)

Participant	Parameter (α)
1	0.27 (±0.04)
2	0.27 (±0.05)
3	0.35 (±0.04)
4	0.06 (±0.04)
5	0.13 (±0.03)
6	0.33 (±0.04)
7	0.58 (±0.02)
8	0.21 (±0.02)

Data are reported as the average (±SD).

**Table 3:** Statistical table

	Data structure	Type of test	Power
PSE comparison(Fig. 2D–F)	PSE derived from psychometric fit	Paired *t* test	D: −0.083:−0.052:−0.021E: −0.055:−0.028:−0.001F: −0.037:−0.021:−0.007
Predicted vs measured PSE	PSE from psychometric fit and PSE based on integration model	Pearson correlation	Power: 100%
Precision depends on PSE	PSE and precision from psychometric fit	Pearson correlation	Power: 100%
CP test	CP based on ROC analysis	*t* test against 0.5	0.51:0.54:0.57

For the paired *t* test we report the 95% confidence interval for the difference distribution and the mean difference. For the correlations we report the Power as computed for a an alpha value of 0.05 and 48 data points. For the *t* test, we report the 95% confidence interval and the mean of the data.

The psychometric curves of the example participant in Figure 2 show that precision ($\sigma^{-2}$ in Eq. 1) decreases as the difference between translated distance in the reference and probe intervals (i.e., the bias) increases. To further investigate this effect, Figure 5 shows a linear relation (*R*^2^ = 0.65, *p* < 0.01) between the bias and precision across all participants and conditions. This effect, which follows Weber’s perceptual law (Fechner, 1860), is consistent with the signal dependence of (discrimination) precision that has been shown recently for vertical self-motion (Nesti et al., 2014).

Thus, the results of the first experiment suggest a direct influence of the eye-movement magnitude on the perception of self-motion, even in complete darkness. However, a trial in experiment 1 always contained at least one translation with a visually constrained fixation point (either world or body fixed). In experiment 2, we removed all fixation constraints and left the eyes free to move during both translation intervals. Subjects again had to indicate whether the second translation was longer or shorter than the first.

**Figure 6.**
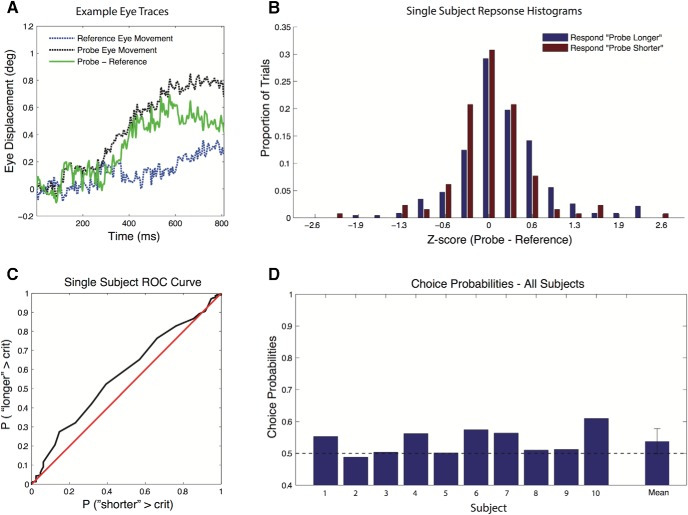
Choice probabilities derived from trial-by-trial normalized eye-displacement differences. ***A***, Exemplar eye traces from two consecutive body translations of the same magnitude. ***B***, All trials were collapsed after *z*-scoring the eye-displacement differences per condition. Trials were split based on the subject perceiving the second translation as being longer or shorter. ***C***, ROC curve based on the data in ***B***. ***D***, Choice probabilities for the individual subjects derived from their ROC curves, showing a significant eye-displacement effect on choice probability across subjects (*p* = 0.018).

We conducted an ROC analysis on the within-trial normalized eye-movement magnitude differences (Δeye), separated into two distributions based on the participants’ responses (see Materials and Methods). Figure 6*A* shows the eye traces from an exemplar trial. Although the underlying whole-body translations were the same for the two traces, the resulting eye movements are clearly different. Figure 6*B* shows the normalized eye-movement difference distributions (Δeye) split based on the subject’s perception of the second displacement being “longer” or “shorter.” From these two distributions, we constructed the ROC curve in Figure 6*C*. The vertical axis depicts the proportion of the Δeye distribution of the longer trials being smaller than the criterion value, and the horizontal axis depicts the proportion of the Δeye distribution of the shorter responses being smaller than the criterion value. If the two distributions are separable based on Δeye, the curve should be systematically above the identity line, as is clear for this subject. Also, across all subjects the CP is above the chance level (Fig. 6*D*; CP = 0.53, *p* = 0.018, single sided), suggesting that the difference in eye displacement is a good predictor of the participants’ perceived difference between the two translations.

## Discussion

We investigated the contribution of eye movements to the perception of passively induced self-motion. Experiments were performed in the absence of full-field optic flow to eliminate the contribution of this visual motion signal. In experiment 1, perception of self-motion was compared across the following three fixation types: during free fixation, the fixation target was extinguished before the movement, while during the world and body condition fixation targets remained stable relative to the world and body, respectively. Our results show that self-motion is underestimated during body fixation (in which the eyes remain stationary) compared with world fixation (in which the eyes move to maintain fixation). Self-motion perception further mimicked the pattern of eye movements during free fixation, which show a nonunity gain with excursions in between body-fixation and world-fixation conditions. To characterize the eye-movement contribution in proportion to the vestibular contribution, we first fitted a single-parameter model to the perceptual responses for the body versus world comparison conditions. We then validated this model independently by demonstrating a correlation between eye movements and self-motion perception during free-fixation conditions. Results from experiment 1 suggest that extraretinal eye-movement signals are used as a cue in the perception of self-motion, contributing significantly to the self-motion percept with a weight of ∼25%, even in the absence of optic flow.

In experiment, 2 we further substantiated this notion that eye movements contribute to self-motion perception. We derived the choice probability from the VOR-driven eye movements only and removed any influence of fixation context. Choice probabilities were above chance level, again suggesting that extraretinal eye-movement signals contribute to the perception of self-motion magnitude.

It is surprising that an influence of eye movements can be observed even for body-stationary fixations, during which the stationary eye-movement signal is clearly in conflict with the nonzero vestibular signal. While this demonstrates the strength of the assumption that fixation targets are world stationary, it raises the question of how reliable this assumption is. Simultaneous recording of angular head and eye movements during natural behavior reveals that ∼80% of eye movements can be classified as compensatory (i.e., eye movements directed opposite to head movement and therefore consistent with maintenance of world-fixed fixation; Einhäuser et al., 2007). Similarly, other studies have shown that world-stationary fixations are common for many everyday activities, ranging from making a cup of tea (Hayhoe and Ballard, 2014) to driving a car (Land and Lee, 1994), to walking (Foulsham et al., 2011) and even reaching, where people tend to look at the source and destination of the object, but not at the hand (Flanagan and Johansson, 2003). Because world-stationary fixations are so common, the natural world statistics imply that self-motion and eye movements are highly correlated, thus making eye movements a fairly reliable cue for self-motion.

Even when fixation is not world fixed, eye-movement signals are combined with optic flow signals to yield realistic self-motion estimates (Royden et al., 1992; van den Berg and Beintema, 2000). During world-fixed fixation, the eyes move to compensate for body translation, thereby reducing the optic flow component in the retinal signal. The self-motion estimate will therefore be driven predominantly by the eye-movement signal. On the other hand, when fixating a body-fixed target, eye movements are minimal and optic flow is maximal, such that perceived self-motion will be driven predominantly by the optic flow signal itself. Because our experiment was performed in darkness, this optic flow signal was absent in the body-fixed condition, which can explain why self-motion was underestimated.

During body and world fixation, eye movements are driven by retinal slip of the fixation target. However, in the free-fixation conditions, retinal slip is not available, and the resulting eye movements resemble the LVOR, in that the gain relative to world fixation was ∼0.4 (Fig. 3*B*; Ramat and Zee, 2003). This reflex is thought to be driven by a double integration of the vestibular signal, converting the head acceleration signal from the otoliths to eye position (Green et al., 2007; Walker et al., 2010). If eye movements during free fixation are in fact vestibularly driven, then the combination of this eye-movement signal with the vestibular signal itself seems redundant. However, such a combination could reflect a strategy to reduce noise. Both the direct (vestibular) and indirect (LVOR) signals depend on integration of the linear acceleration signal and may be corrupted by independent noise sources. Combining them in a statistically optimal fashion will decrease the noise level toward the noise level of the original source signal (Faisal et al., 2008; Clemens et al., 2011; Fetsch et al., 2013). The consequence of this integration will be a reduced self-motion estimate when the gain of the LVOR is <1, as we observed in the free condition in experiment 1. This also explains why biases in self-motion estimates are correlated with free eye-movement magnitude differences in experiment 2. We hypothesize that the adverse consequences of this seemingly inflexible arrangement, which may be learned or innate (Nardini et al. 2008), are minimal under natural conditions because eye movements and self-motion are highly correlated, and because eye movements are most often accompanied by veridical optic flow cues to self-motion.

### Alternative interpretations

In the above, we suggest that eye movements themselves drive the perception of self-motion. However, it is conceivable that a common correlate of eye movements, such as attention or visual motion influenced our results. Guedry and Harris (1963) reported a substantial underestimation of displacement when their observers watched a small body-fixed target compared with displacements in the dark. They attributed their findings to an attentional shift from judgments of body displacement in the dark to judgments of target displacement in the fixation condition. We favor an explanation by eye movements. In their study, it is likely that the VOR caused eye movements to occur during the translations in darkness. If these movements were used to augment self-motion perception, then the perception of such translations would be overestimated compared with translations made without eye movements (e.g., when fixating a body-fixed target). Because Guedry and Harris (1963) neither recorded nor explicitly manipulated eye movements, they were not able to unveil their explicit role. Likewise, we did not manipulate attentional processes (Kitazaki and Sato, 2003), so we cannot completely exclude the possibility that they play a role.

Others have reported errors in the disambiguation of self-motion and object-motion. Examples include the perceived motion of head-fixed visual targets in the direction of angular acceleration (the oculogyral illusion), which has been related to the retinal slip present and the magnitude of suppression of the VOR eye movements (Carriot et al. 2011). Similarly, during linear accelerations, observers perceive a body-fixed stimulus as displaced in the direction of acceleration (the oculogravic illusion; Graybiel, 1952), while a truly world-stationary stimulus seems to move in a direction opposite to the observer’s motion (Dyde and Harris, 2008). In other words, for a stimulus to appear stable in the world it needs to move consistently in the same direction as the observer (Dyde and Harris, 2008). Such disambiguation errors could cause the effects we observed, if movement of the fixation point relative to the observer were always attributed to self-motion. That is, with world fixation, the perceived translation of the observer from the target is relatively overestimated, while with body fixation the perceived translation is underestimated, as we observed. However, such attribution errors cannot account for the effects in the free conditions in the two experiments, because no fixation point was visible and no attribution was required. In the experiment with free gaze, we demonstrate that eye movements by themselves, occurring in the absence of visual tracking and other external cues, are correlated with the perception of self-motion.

### Implications for other studies

Many previous self-motion studies have used a body-fixed fixation point to control for eye movement-related effects. Our results suggest, however, that using a body-fixed fixation point causes the underestimation of self-motion. For example, Li et al. (2005) investigated spatial updating across lateral translation and found that saccades to updated targets undershot the actual target location. As self-motion perception drives this update, the effects of eye movements on self-motion perception should also influence the updating process. In other words, the observed undershoot could be due to the underestimation of self-motion caused by the body-fixed fixation point. Another example is a study of the perception of vertical object motion during lateral translation (Dokka et al., 2015). This study reports incomplete compensation for self-motion when judging the deviation from vertical motion of a moving object. This observation could also be due to the underestimation of self-motion induced by the fixation of the body-fixed target.

A moving fixation point is also known to influence self-motion perception, as in the Slalom Illusion (Freeman et al., 2000): observers viewing expanding optic flow while fixating on a target that oscillates from left to right perceive slaloming motion, which is inconsistent with the purely forward motion specified by the expanding optic flow display. However, this observation is consistent with the idea that oculomotor signals are used in estimating self-motion. Additionally, it has been shown that eye movements affect postural sway (Glasauer et al., 2005). Participants performed smooth pursuit eye movements in complete darkness and displayed lateral sway consistent with the stabilization of posture using a self-motion estimate influenced by pursuit eye movements.

Studies conducted to characterize vestibular-only sensitivity are often performed in complete darkness or with closed eyes (Grabherr et al., 2008; MacNeilage et al., 2010a,b; Roditi and Crane, 2012; Valko et al., 2012; Nesti et al., 2014). However, the results of our free-fixation conditions suggest that, even under these circumstances, results could easily be influenced by vestibularly driven eye movements. Overall, we suggest that any study concerned with self-motion processing must consider the possible influence of eye movements.

### Possible neural substrate

This leaves us with the question of where in the brain these effects originate. The locus of our effect is likely to carry both eye-movement and vestibular signals. Prime candidate areas known to carry both vestibular and eye-movement signals are the vestibular nuclei (Henn et al., 1974; Daunton and Thomsen, 1979) and the cerebellum (Waespe et al., 1981). On the other hand, eye movements could influence self-motion perception indirectly via optic flow processing. In particular, cortical areas that carry both vestibular and optic flow signals (which can be modulated by eye movements) include the ventral intraparietal area (Bremmer et al., 2002; Chen et al., 2011), and the dorsal medial superior temporal area (Gu et al., 2008). Future work should reveal how such brain areas, directly or indirectly, merge both vestibular and oculomotor signals into a coherent percept of self-motion.

## Synthesis

Reviewing Editor: Kathryn Jeffery, University College London Decisions are customarily a result of the Reviewing Editor and the peer reviewers coming together and discussing their recommendations until a consensus is reached. When revisions are invited, a fact-based synthesis statement explaining their decision and outlining what is needed to prepare a revision will be listed below. The following reviewer(s) agreed to reveal their identity: Jerome Carriot, Aman Saleem

Overall, the study is interesting and well carried out. Only a few arguments need to be added or discussed to improve the manuscript.

1. A couple of times in the paper the authors hypothesize that vestibular cues and eye movement cues should be additive. However, it is rare that multi-sensory processes serving the same modality (i.e. self-motion perception) will quantitatively change this modality. For example, it has been shown in MSTd that vision and vestibular cues are complementary: when vision is the most reliable cue the system uses vision and if visual cues are less reliable the system uses vestibular cues. If both modalities are available the system uses both to get a more accurate estimation of self motion. However, both cues are not added together to provide an estimation of self-motion that would be bigger than what it is. The same process does not seem to applied in the current experiment. Eye movement cues seem to be falsely interpreted and change the length of the perceived displacement. Thus, the authors could moderate the hypothesis formulation in the introduction section.

2. As authors mentioned in the discussion, a head fixed target could be perceived as moving in the direction of the acceleration. This illusion has been shown to be due to the misinterpretation of the VOR cancellation signal (and the remaining retinal slip). The illusory motion of the target in the direction of the acceleration would be conflicting with the vestibular sensation of movement, which could explain why the motion with the head-fixed target is perceived as shorter than the motion with the world-fixed target. Some further discussion of this issue would be useful.

Abstract: 2AFC needs to be worded in full in the abstract. Also a typo: eye movementS were constrained

Experimental setup - the text reading [setup ref to be inserted] needs replacing

Line 97 pinnea should be pinna

In the discussion of Experiment 1, it is twice suggested that the results indicate a contribution of eye movements to motion estimation: line 327 and line 440. However, an alternative interpretation is that the fixation point acts as a static landmark which can contribute to post hoc self-motion estimation. Experiment 2 helped refute this possibility but the possibility should neverthess be mentioned.

• Figure 2: It would aid the readers if the identity of the ‘single participant’ in panels A,B and C are marked out in panels D,E and F.

• Figure 3: It would be useful if panel A also shows the normalized eye displacement (for example, an additional y-axis on the right). This would be directly relate the results of panel A to panel B.

• In the methods, the authors mention that the ‘catch-up’ eye movements are removed from further analysis (Lines 262-267). It would be nice to see an illustrative example of this procedure.

• The integration of vestibular and visual information could either be inherent in the neural network, or might be learnt with experience (Nardini et al, 2008). It might be useful to discuss this aspect.
